# Locating Forest Management Units Using Remote Sensing and Geostatistical Tools in North-Central Washington, USA

**DOI:** 10.3390/s20092454

**Published:** 2020-04-26

**Authors:** Palaiologos Palaiologou, Maureen Essen, John Hogland, Kostas Kalabokidis

**Affiliations:** 1 Department of Geography, University of the Aegean, 81100 Mytilene, Greece; 2 USDA Forest Service, Rocky Mountain Research Station, Missoula, MT 59801, USA

**Keywords:** forest treatments, change detection, Random Forests, principal component analysis, Sentinel-2, Satellite Image Time-Series, RMRS Raster Utility

## Abstract

In this study, we share an approach to locate and map forest management units with high accuracy and with relatively rapid turnaround. Our study area consists of private, state, and federal land holdings that cover four counties in North-Central Washington, USA (Kittitas, Okanogan, Chelan and Douglas). This area has a rich history of landscape change caused by frequent wildfires, insect attacks, disease outbreaks, and forest management practices, which is only partially documented across ownerships in an inconsistent fashion. To consistently quantify forest management activities for the entire study area, we leveraged Sentinel-2 satellite imagery, LANDFIRE existing vegetation types and disturbances, monitoring trends in burn severity fire perimeters, and Landsat 8 Burned Area products. Within our methodology, Sentinel-2 images were collected and transformed to orthogonal land cover change difference and ratio metrics using principal component analyses. In addition, the Normalized Difference Vegetation Index and the Relativized Burn Ratio index were estimated. These variables were used as predictors in Random Forests machine learning classification models. Known locations of forest treatment units were used to create samples to train the Random Forests models to estimate where changes in forest structure occurred between the years of 2016 and 2019. We visually inspected each derived polygon to manually assign one treatment class, either clearcut or thinning. Landsat 8 Burned Area products were used to derive prescribed fire units for the same period. The bulk of analyses were performed using the RMRS Raster Utility toolbar that facilitated spatial, statistical, and machine learning tools, while significantly reducing the required processing time and storage space associated with analyzing these large datasets. The results were combined with existing LANDFIRE vegetation disturbance and forest treatment data to create a 21-year dataset (1999–2019) for the study area.

## 1. Introduction

In the USA, the annual average number of large fires has tripled and the average fire size increased by at least six times since the 1970s [1]. While dramatic, this increase in wildfire activity does not fully compensate for the fire deficit seen across forested landscapes in the USA [2]. The fire deficit, described as the difference between the historical rate of burning and the current rate of fire frequency plus mechanical treatments, in large contributes to higher density forests with larger quantities of fuels [2]. Owing to this circumstance, private, state, and Federal agencies within the USA face ever-increasing fire suppression costs. For example, in 2017, USA federal agencies alone spent approximately USD 2.9 billion in fire suppression [3], and are projected to see a growth in the 10-year average fire suppression cost of nearly USD 1.8 billion through 2025 [4]. This increase in wildfire frequency, size, suppression costs, and other factors has helped promote fuel management in both wildlands and home ignition zones inside the Wildland-Urban Interface (or in both during cross-boundary projects) as an available short term option compared to suppression alone to cope with wildfire related issues [5,6,7].

Despite disagreements on how, when and where those treatments should be applied [5,8,9,10], fuel management is a common practice among private, state, tribal, and federal forest managers in the USA. Broadly, forest fuel treatments alter key characteristics of forested ecosystems and modify fuel arrangements and compositions. Forest management techniques used to reduce fuels include mechanical treatments (e.g., clearcut, mastication, thinning), prescribed or managed fire, grazing, and chemical or biological applications [11]. These approaches can be used as standalone treatments or combined in various ways to reduce fuel loads, e.g., thinning followed by a prescribed fire [12].

Rational for these types of treatments depend on social and ecological objectives and are not limited to reducing forest fuels to protect values-at-risk, e.g., homes, agricultural land, powerlines, but also to support timber economies or achieve ecological objectives such as restoring watersheds or improving forest health. For example, forest managers may aim to address the effects of long-term fire suppression by completing fuel treatments to change wildfire frequency and behavior [13,14]. Similarly, treatments may aim to address climate change by altering the carbon balance [15].

The range of objectives and outcomes of forest treatments underscore the need to identify the temporal and spatial arrangement of past treatments across forested landscapes to inform future management options and to study historic forest management approaches. In the USA, the Landscape Fire and Resource Management Planning Tools (LANDFIRE program) [16] provides a complete set of natural disturbance and treatment geo-spatial data covering the period 1999–2016. This data are organized in polygons by treatment type and year of occurrence or observation. While data provided by the LANDFIRE program are valuable and have been used by researchers and managers for many years, they require a significant investment of resources over a long period of time to produce. For example, treatment and disturbance data are collected from disparate sources, including federal, state, local and private organizations and later processed into a variety of data products. As a result, there is a lag time associated with producing datasets and data for 2017–2019 will not be available for years to come. In the meantime, data from 1999–2016 are freely available. While invaluable to forest managers, these types of data outside the USA are largely unavailable or with significant temporal and spatial gaps. To address the temporal gap in treatment data for the USA and to reduce the cost of updating existing datasets, we describe an analytical approach using freely available satellite imagery to locate forest treatments (i.e., clearcut, thinning, prescribed fire) between 2016 and 2019 in a four-county study area in Washington, USA. 

Previous studies showed that forest harvest patterns can be readily detected with high accuracy using Landsat imagery, mainly because this type of cover change in forested lands is expressed as a large spectral contrast in a temporal image data set [17,18,19]. Thinning cuttings can also be detected from the changes in reflectance of multispectral sensors [20,21,22,23,24]. Remote sensing time-series products, combined with statistical methods, novel algorithms and classification approaches have been previously applied to map deforestation and forest degradation in tropical ecosystems [25,26,27,28,29], wildfires and clearcut harvest disturbances in boreal forests [30], disturbance history including clearcuts in northern America’s forests [31,32,33,34,35], and insect attacks and disease outbreaks recording their impacts on defoliation and other forest properties [36,37,38].

The primary goal of this study was to geospatially identify mechanical fuel treatments to build a retrospective view of selected forest management treatments that can be used to inform future forest management decisions. This methodology, paired with existing LANDFIRE data and Landsat burned area estimates [39], yielded a complete 21-year dataset (1999–2019) for the study area in North-Central Washington, USA. A secondary goal was to identify a repeatable method to locate forest treatments over large spatial extents using satellite imagery and machine learning processes. Accordingly, we hypothesize that there is a detectable spectral difference between mechanically treated and untreated spatial extents, and that differences can be categorized into a subset of mechanical treatments of interest.

## 2. Materials and Methods 

### 2.1. Study Area

**Figure 1:**
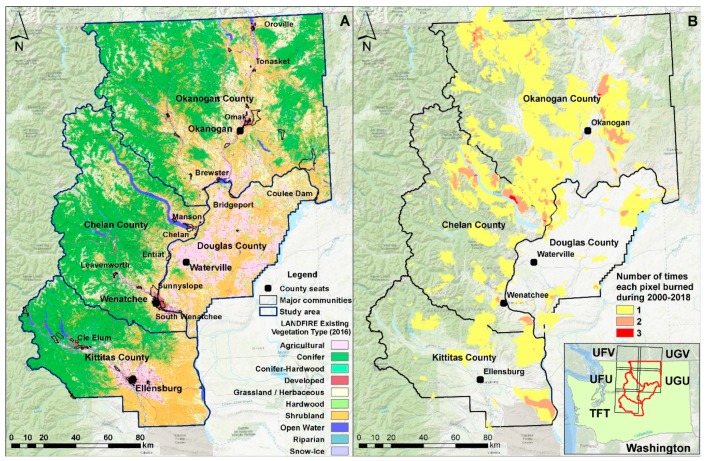
(**A**) Existing vegetation types of 2016 (data source: LANDFIRE); (**B**) Number of times each area was burned during the period of 2000–2018 (data source: LANDFIRE disturbances [16] and Monitoring Trends in Burn Severity [42]); Inset map shows the location of the four counties over the state of Washington, while black rectangles depict the boundary of each of the five Sentinel-2 tiles.

**Figure 2:**
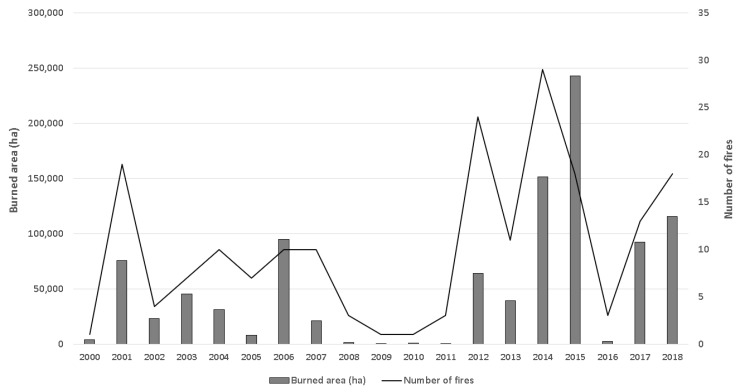
Burned area and number of fire events greater than 100 ha for the period of 2000–2018. Data source: LANDFIRE disturbances [16] and Monitoring Trends in Burn Severity [42].

Our study area comprises 3.2 million hectares (ha) and four counties in North-Central Washington, USA: Kittitas, Okanogan, Chelan and Douglas Counties (Figure 1A). We selected this study area due to evidence from previous studies [40] and reports [3,41] that revealed high rates of fire transmission across a multitude of ownerships and jurisdictions and the level of exposure of nearby communities to this wildfire hazard (Figure 1B). In addition, between 2000 and 2018, more than one million ha burned inside the four counties, including 150,000 ha in 2014 and 240,000 ha in 2015 (Figure 2).

Okanogan County is the largest county in the state (1.37 million ha), with a population of 42,132 (2018 census). In July 2014, the Carlton Complex wildfire burned over 100,000 ha in Okanogan County. Other major fire events include the 2017 Diamond Creek Fire (52,000 ha), the Okanogan Complex Fires during 2015 (123,341 ha), the Tripod Complex Fire during 2006 (71,000 ha) and the Okanagan Mountain Park Fire during 2003 (26,000 ha). Kittitas County has a population of 47,364 in an area of 595,000 ha. In August 2017, the Jolly Mountain Fire burned 15,000 ha. A few years earlier (2012), the Taylor Bridge Fire and the Table Mountain Fire burned 10,000 ha and 17,000 ha respectively. Chelan County covers an area of 757,000 ha, with a population of 77,036 people. Some noteworthy fires include the Uno Peak Fire (3500 ha in August 2017), the Chelan Complex Fires (36,000 ha) and the Wolverine Fire (26,500 ha) during 2015, and the Deep Harbor Fire (11,500 ha) during 2004. Finally, Douglas County covers an area of 471,000 ha with a population of 42,907 people, in mostly rural and agricultural areas. Among the most important fires are the Barker Canyon Complex Fire during 2012 (33,000 ha) and the Douglas County Complex Fires during 2015 (9000 ha).

### 2.2. Satellite Images and Preprocessing

**Figure 3:**
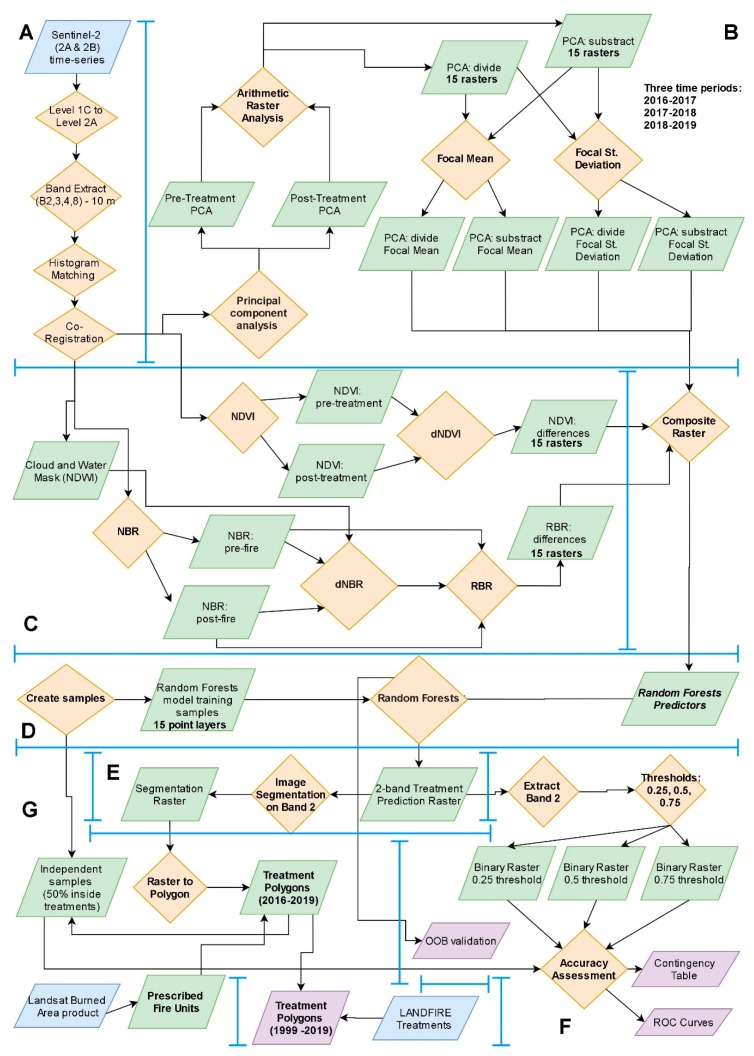
Methodological flowchart of the proposed solution to detect fuel treatments. Blue rectangle: Input; Orange diamond: Process; Green rectangle: Intermediate Dataset; Purple rectangle: Output. (**A**): Satellite Images and Preprocessing; (**B**): Principal Component Analyses; (**C**): Vegetation Indices; (**D**): Random Forests; (**E**): Image Segmentation; (**F**): Accuracy Assessment; (**G**): Fuel Treatment Polygons.

In Figure 3, the flow of the proposed method is illustrated that is detailed in the following Sections. Spectral reflectance was recorded and converted into images by the Sentinel-2 (2A and 2B) Multispectral Instrument, operated by the European Space Agency (ESA) under the Copernicus program [43]. The Sentinel-2 images sample 13 spectral bands: four bands at 10 m, six bands at 20 m and three bands at 60 m spatial resolution. To ensure similar atmospheric conditions and radiometric properties, we acquired orthorectified images between the years of 2016 and 2019 (4 years) from late summer or early autumn (Table 1) with minimal cloud and smoke cover (<10%) that were derived from a single descending orbit (i.e., track 13). For 2018, October was the only month with available images having less than 10% cloud cover. The study area was covered by five tiles of the same track (tft, ufu, ugu, ufv and ugv), with the last two extending to Canada (Figure 1B). A sixth tile, the tgt, which covers half of the Douglas Country and a small part of Kittitas County, was not included in the analysis since it is mostly covered by agricultural lands or grasslands that historically do not receive mechanical fuel treatments. All images were processed to Level-1C (Top of Atmosphere reflectance) except those from 2019 that were already pre-processed to Level-2A (surface reflectance) [44]. We used the official ESA software called SNAP to convert Level-1C products to Level-2A with the Sen2Cor v2.8 package, also applying Cirrus cloud corrections [45]. A sun angle grid was computed by regularly down-sampling the Level-1C tiles, while the solar zenith and azimuth angles were specified for the tile corners with a subsequent bilinear interpolation across the scene. For each image, we extracted the bands two, three, four, and eight, to be used as baseline image datasets based on spatial resolution criteria (Blue, 490 nm; Green, 560 nm; Red, 665 nm, and Near Infrared, 842 nm; grain size 10 m). In addition, band 12 (Short-Wave Infrared, 2190 nm, grain size 20 m) was upsampled to 10 m resolution with Bilinear interpolation, but only to be used as intermediate dataset for the estimation of Relativized Burn Ratio index (see next Sections for details). Previous studies found that the short-wave infrared portion of the electromagnetic spectrum is effective at separating fires and clearcut harvests [30].

Many images that captured the same spatial area across years were slightly different due to the sun angle or atmospheric effects at the time each image was taken. To address this, we applied histogram matching with ERDAS Imagine [46]. We started by matching each 2016 image with corresponding 2017 images using all four bands. Then, we used the resulting images of 2017 to match with 2018 images and resulting 2018 images to match 2019 for each Sentinel tile. Sentinel-2 products for 2016 and 2017, before the ESA Global Reference Image [47] incorporation into the processing, can be misregistered by more than one 10-m pixel [48,49]. To reduce misregistration error, we used the AutoSync Workstation of ERDAS Imagine 2014 to co-register the pixels of the two images for two sequential years, starting with 2016 and 2017. We used the NIR band for the generation of the automatic point measurement algorithm, which creates a large set of random points between the two images. We removed all random points with error > 0.5 pixels and georeferenced the image with the Cubic Convolution resample method. The sum of these steps resulted in a set of comparable raster-based data for the years from 2016 to 2019.

### 2.3. Post-Processing of the 4-Band Images

Analyzing raster datasets using multiple spatial operations and creating and reading new raster datasets at each step comes at a high processing and storage price, often limiting the types of analyses that can be performed. To address this challenge, we used the Rocky Mountain Research Station (RMRS) Raster Utility toolbar, a product of the US Forest Service RMRS [50]. The RMRS Raster Utility extension provides a modeling framework called Function Modeling that utilizes lazy or delayed reading techniques to process raster functions without creating intermediate outputs, using function raster datasets [51]. The use of Function Modeling can significantly reduce both the processing time and storage requirements associated with spatial modeling of raster datasets.

Using Raster Utility singular vector decomposition principal component analyses (PCA), we created orthogonal principal component raster surfaces for each Sentinel-2 image. PCA is a mathematical procedure for spectral enhancement that uses orthogonal transformation to convert correlated variables into a set of linearly uncorrelated bands [52]. For our study, we estimated the four principal components (PC) for each of the 20 four-band Sentinel-2 images (five tiles for each year, with four principal components each).

To build predictor variables, we performed two separate spatial calculations for each pair of the PC surfaces (pre- and post-treatment year), i.e., an arithmetic subtraction (*PCA_sub_*, Equation (1)) and a division (*PCA_div_*, Equation (2)) using the Arithmetic Analysis command with Raster Utility as follows:

(1) $$PCA_{sub}=\;PCA_{iw}-\;PCA_{in}$$

(2) $$PCA_{div}=\;PCA_{in}/PCA_{iw}$$

where, *i* is the *i*th PC band cell score of the post-treatment year *w* or of the pre-treatment year *n*.

As a result, for each tile, two new four-band raster surfaces were produced for the combination of each pre- and post-treatment year (three time periods, i.e., 2016–2017, 2017–2018, 2018–2019), one from the subtraction and one from the division (a total of 30 rasters that were handled as intermediate data). It is important to note that these rasters were not physically stored as files, since Raster Utility allowed the on-the-fly processing of them.

Next, we performed a Focal Analysis (FA) for each band of PCA_sub_ and PCA_div_ raster surfaces that calculated the mean and standard deviation (STD) within a 3X3 pixel-sized moving window. This process produced 60 additional intermediate raster surfaces, i.e., 3 time periods X 5 tiles X 2 FA rasters for PCA_sub_ (mean and STD) X 2 FA rasters for PCA_div_ (mean and STD).

The Relativized Burn Ratio (RBR) [53] was computed with SNAP. After applying a cloud and water mask on the images of each period, we computed the normalized burn ratio (NBR) from bands 8 (Near Infrared, NIR) and 12 (Short-Wave Infrared, SWIR) (Equation (3)), and then the delta Normalized Burn Ratio (dNBR) (Equation (4)). The dNBR is an absolute difference that can present problems in areas with low pre-fire vegetation cover, where the absolute change between pre-fire and post-fire NBR is small. RBR is more advantageous in such cases [53], computed with Equation (5). The Normalized Difference Vegetation Index (NDVI) was computed with the Raster Utility (Equation (6)).

(3) $$NBR=\;\frac{NIR-SWIR}{NIR+SWIR}$$

(4) $$dNBR=NBR_{pre-fire}-NBR_{post-fire}$$

(5) $$RBR=\left(\frac{dNBR}{\left(NBR_{pre-fire}+1.001\right)}\right)$$

(6) $$NDVI\;=\;\frac{NIR-Red}{NIR+Red}$$

We then combined the NDVI, the RBR, and the four intermediate FA raster surfaces of each tile into one multiband raster surface using the Composite Raster tool within the Raster Utility. The resulting final predictor raster surface for each tile of each time period had 18 bands (predictor variables) consisting of: 4 for focal mean of PCA_sub_, 4 for focal STD of PCA_sub_, 4 for focal mean of PCA_div_, 4 for focal STD of PCA_div_, the RBR and the NDVI. We then applied the ArcGIS command of “Band Collection Statistics” to compute covariance and correlation matrices across all pixels and among all predictor raster bands of each tile. Where predictor raster bands were highly correlated (>0.75 or <−0.75), we flagged the band(s) with the lowest rank and removed them as inputs to the Random Forests analysis (e.g., if raster band 1 was correlated with raster band 10 we flagged the latter).

### 2.4. Creating Training Samples

First, we used the Existing Vegetation Type (EVT) dataset of the latest LANDFIRE data (circa 2016) to mask non-forest pixels (i.e., pixels characterized as grasslands, agricultural lands, developed areas, snow, ice, riparian, sparsely vegetated, quarries, water, exotic herbaceous and roads). LANDFIRE’s EVT represents the current distribution of the terrestrial ecological systems classification, defined as a group of plant community types (associations) that tend to co-occur within landscapes with similar ecological processes, substrates, and/or environmental gradients.

**Figure 4:**
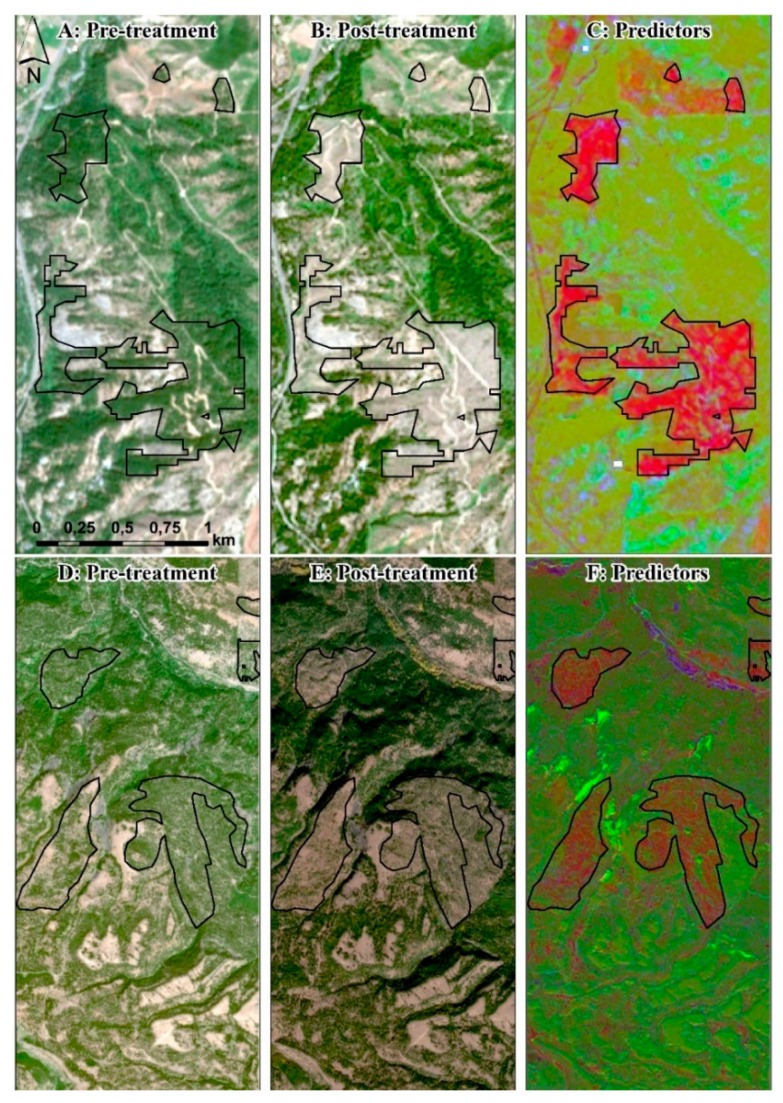
Initial identification of vegetation mechanical treatment units for: (a) clearcut (panels **A**–**C**); and (b) thinning (panels **D**–**F**). (**A**,**D**): Pre-treatment Sentinel-2 image on natural color; (**B**,**E**): post-treatment Sentinel-2 image on natural color; (**C**,**F**): predictor variables (Red: NDVI; Green: RBR; Blue: first component of focal STD for PCA_sub_). The black outline denotes the final treatment perimeter, derived from the next steps of the analyses.

To create Random Forests training datasets, we randomly selected 500 locations inside non-masked forested areas (e.g., Figure 4A,D). We then visually examined each point over two sequential years of Sentinel-2 images to identify areas as either treatment or non-treatments units (1 and 0, respectively). Since not all forest treatments could be visually identified in this way (e.g., prescribed fires, or pile burns), we focused on identifying points associated with potential mechanical treatments, more specifically clearcut and thinning (Figure 4B,E respectively). To aid in this effort, we visualized the predictors raster with a band combination of Red: NDVI; Green: RBR; Blue: first component of focal STD for PCA_sub_ (see Figure 4C,F). In addition, we further verified each point’s assigned value using the Global Forest Change 2000–2018 database (for the years 2016–2018) [54], which identifies locations of stand-replacement disturbances or a change from a forest to non-forest state. Finally, the proximity of sampling points to known past fuel treatments increased the certainty of whether that point was inside a treatment, minimizing the risk of using points that fall inside mortality, insect attacked or weather damaged stands.

As expected, forest treatments occupy a very small part of the total landscape, resulting in only very few of the 500 randomly located points falling inside treated areas. To capture the large variability of conditions outside the treatment locations, we defined a rule to achieve a ratio within each training dataset of approximately 30% treatment points (classified as 1) and 70% non-treatment points (classified as 0). While an imbalance in the training data is a known statistical issue when performing classification [55,56], decision trees tend to perform well on imbalanced datasets when a substantial number of observations fall within each category and can be used to split class labels within predictor variables space.

To ensure that the 30% treatment vs. 70% non-treatment threshold was met, we manually added locations in the training sample inside visually identifiable treated lands, or randomly selected and moved some of the 500 original points flagged as non-treatments to locations inside treated lands. On average, across all tiles and through all the three periods, approximately 450 locations were identified as non-treatments and about 200 locations where identified as treatments. Finally, for each training sample location, the predictor raster surface value of each band was extracted. In total, these procedures yielded one training data set for each of the 15 pairs of pre- and post-treatment year predictor raster surfaces.

### 2.5. Random Forests

Training datasets were used with a Random Forests algorithm [57] to create a suite of classification models used to classify each predictor surface pixel into one of two values, treatment (value = 1) or no treatment (value = 0). Random Forests were applied on the set of predictors for each of the 15 pairs of pre- and post-treatment year raster surfaces. Random Forests is an ensemble machine learning method that fits many classification or regression tree models to random subsets of the input data and uses the combined result (the forest) for prediction. A principal feature of Random Forests is its ability to calculate and estimate the importance of each predictor variable in modeling the response variable without making assumptions about the distribution of the data.

During model training, we used only uncorrelated predictor variables (see Section 2.3) and specified the number of predictor variables randomly selected at each tree node as the square root of the total number of potential uncorrelated predictor variables (rounded down when the square root was not a whole number). We set the number of trees to 100 and a train ratio of 0.66. As a result, for each tree a random subset of the 66% of all sample locations were used to train the model and the remaining 34% were used as an “out-of-bag” (OOB) dataset to perform an independent tree-based accuracy assessment of the model. This accuracy assessment calculated the Root Mean Square Error (RMSE—error when estimating posterior probabilities), the average relative error (average relative error when estimating posterior probability of belonging to the correct class) and the relative classification error (percent of incorrectly classified cases).

We used a weighted voting approach and built a two-band raster surfaces using the Raster Utility tools. Each band’s cell value within the two-band raster output corresponds to the proportion of times a given class was predicted as a given label (the ratio of pixels classified as treated vs. not-treated). Raster surface bands correspond to non-treatment (band 1) and treatment labels (band 2). To identify the homogenous grouping of treatment areas, we extracted only the treatments raster (band 2) and used it as an input to ArcGIS’s Mean Shift Segmentation algorithm.

### 2.6. Image Segmentation

The Mean Shift Segmentation command of ArcGIS works by controlling the amount of spatial and spectral smoothing used to derive features (or segments) of interest. In our case, we were interested in this operation to further hone the identification of forest treatments on the landscape by grouping cells into clusters of similar spectral values (in our case, the proportion of times a given class was predicted by Random Forests as treated). To discriminate between features having similar spectral characteristics, we set the spectral detail parameter to the highest value (i.e., 20) while keeping spatial details at a moderate level (i.e., 10). The minimum segment size was set at 10 pixels to filter out smaller groups of pixels. The image segmentation created a new raster for each of the 15 Random Forests resulting raster surfaces, where higher segment values were most likely to correspond to forest treatments.

### 2.7. Treatment Polygons

Since we already had identified some prominent forest treatment units over the landscape during the creation of model training samples (Section 2.4), we used them to define threshold values within the segmented raster surface to isolate pixels of treatments that could not be easily traced over the landscape. We converted each of the 15 segmentation raster surfaces (5 for each time period) into a polygon layer by classifying all values above the threshold as treatments. While thresholding helped to limit the number treatment polygons identified, there appeared to be a large number of false positive regions using thresholding alone. Additionally, vectorization process introduced noise in the form of slivers.

To address these issues, we first identified all slivers by selected polygons with area smaller than 1 ha and deleted them from our potential treatment areas. Next, we removed all polygons intersecting wildfire perimeters of the target year. For example, in the target year of 2017, all spectrally identified treatment units intersecting the boundary of recorded wildfire perimeters (retrieved from the Wildfire Decision Support System/WFDSS databases [58]) that occurred up to the end of August 2017 (satellite image acquisition date) were removed. Then, we deleted all polygons falling on cloudy or hazy or shadowed or snow-covered areas, and all polygons on high-elevation areas or mountain peaks without road access and no evidence of neighboring past forest treatments from the LANDFIRE databases. Finally, we deleted all polygons falling in agricultural lands or other non-forested areas that the LANDFIRE EVT mask failed to capture. After removing these highly probable false positive treatment polygons from our potential treatment areas, the number of identified treatment polygons for each pre- and post-treatment combination period was approximately 500 distinct polygons.

Next, we visually inspected each of the almost 500 polygons over each pair of satellite images (true-color band combination) to a) ensure that they were true treatment units, and b) label each polygon with its potential treatment type. The complete removal of trees from a unit was labelled as clearcut, and units with the existence of standing trees, but in lower density compared to the pre-treatment year’s satellite image, were labelled as “thinning”. This process was the most time-consuming of the proposed methodology and required multiple iterations of editing to capture all treatments correctly.

### 2.8. Independent Accuracy Assessment

Although the Random Forests approach provided a set of accuracy assessment measures to estimate model accuracy with the OOB samples, we conducted an additional accuracy assessment with new datasets not used during the Random Forests modelling to further ensure the validity of our results and account for any potential influence from the imbalance in the training data (see Section 2.4).

First, we used three different thresholds on the band 2 (see Section 2.5) of the Random Forests output rasters (0.25, 0.5 and 0.75) to create new binary raster surfaces (merging all 5 tiles for each pre- and post-treatment time period, i.e., three binary rasters covering the entire study area for each period). Pixels with a value higher than the threshold were characterized as treatments and assigned a value of 1. We used the treatment polygons created in Section 2.7 (i.e., very high probability of being true forest treatment polygons) to create a random sample of 500 locations within them (value of 1). An additional 500 locations were located inside non-treatment areas, i.e., outside treatment polygons (value of 0). We repeated this process for each pre- and post-treatment time period (3000 sample locations in total). For each random location, we retrieved the corresponding modelled value (either 1 or 0) from the three binary raster surfaces and performed an accuracy assessment using the Raster Utility Accuracy Assessment tool. This tool constructs a contingency table (in our case a 2 × 2 matrix) depicting the relationship between the actual values and the modeled classifications values with a set of statistics that can be used to assess classification accuracy (chi-square, overall accuracy and standard error).

Finally, we built one Receiver Operating Characteristics (ROC) curve for each pre- and post-treatment time period using the same 1000 sample locations of each period. Τhis time, instead of using a hard classification of 1 and 0 for only three thresholds, we appended the Random Forests band 2 value ranging from 0.0 to 1.0. Using these ROC curve plots and Area Under Curve (AUC) statistics, we assessed the sensitivity vs. false positives of the entire range of Random Forests proportions as derived from our classifications for the random sample locations.

### 2.9. Prescribed Fire Units

To identify prescribed fire treatments within our classified treatment areas, we used the Landsat Burned Area product [39]. Landsat Burned Area products are only created for images with a RMSE less than 10 m and cloud cover less than 80%. For each year (2017, 2018 and 2019), we retrieved all the Burned Area products from the Landsat 8 OLI satellite that had cloud cover less than 50%, for all the dates within that year. From the Burned Area product, we selected cell values equal to 1 depicting burned pixels and created a binary burned raster surface (1 = burned, 0 = unburned). We then combined each binary burned raster surface to create one merged raster for each of the three years. After converting burned pixels into a new polygon layer, we deleted all slivers (polygons <1 ha) and all polygons falling within a known and mapped wildfire perimeter (retrieved from WFDSS). In addition, we deleted all polygons falling in agricultural and non-forested lands, as defined by the LANDFIRE EVT mask. The remaining burned area polygons had a very high chance of being prescribed fire units in forested lands.

### 2.10. Integration with LANDFIRE Treatments

To populate our treatment polygons with pre 2017 data we used the LANDFIRE Public Events geodatabase (circa. 2014) and the Model Ready Events feature classes that include natural disturbance and vegetation/fuel treatment data. These polygon data characterized treatment event types (i.e., clearcut, harvesting, thinning, prescribed fire, etc.) between 1999 and 2014. Data were collected by LANDFIRE from disparate sources including, public federal, state, and local, agencies and private organizations and subsequently evaluated for inclusion in the LANDFIRE Public Events geodatabase. After identifying overlap between polygons within the same year (i.e., exactly the same polygon in terms of area, shape and location), LANDFIRE reduced the data to include only one unique event per year per location in the Public Model Ready Events feature class. The event types are ranked on a list (see Appendix B on readme at [59]) based on their impact on vegetation and/or fuels composition and structure (i.e., development, followed by clearcut, harvesting, thinning, mastication and so on). This list defined which event types were deleted in case of overlaps (the highest rank was kept).

For our purposes, we selected the following event types from the Public Model Ready Events feature class: clearcut, harvesting, thinning, mastication, other mechanical treatment and prescribed fire. We kept two versions of this layer: the original, which was used to create detailed estimates of the treatment types occurred in the area, and a modified one where we removed any overlap of the same polygons (each treatment polygon is unique). For the years 2015 and 2016, we retrieved the raster version of the LANDFIRE Remap and converted those raster surfaces to polygons while also performing some cleaning of the data to remove slivers and small polygons (<1 ha).

Following this conversion, we noted that several treated areas that were visible within the satellite images were not included in the LANDFIRE Public Events database [59]. These treated areas were classified with an “unknown” event type, but they were available only in the raster format of LANDFIRE Disturbance layer. This raster disturbance layer is created with Landsat satellite products and data included in the LANDFIRE Public Event Database and the National LANDFIRE Reference Database LFRDB datasets [60]. LANDFIRE uses the Multi-Index Integrated Change Analysis (MIICA) algorithm [61] to identify areas where disturbances may have occurred (spectral changes between pre- and post-year of Landsat images). During LANDFIRE mapping processes, once all Landsat-detected disturbances were mapped, causality was assigned by intersecting each disturbance pixel with the following datasets ordered by precedence: (1) Monitoring Trends in Burn Severity—MTBS [42], (2) Burned Area Emergency Response—BAER [62], (3) Rapid Assessment of Vegetation Condition after Wildfire—RAVG [63], (4) Public Event Database, and (5) Landsat Burned Area Essential Climate Variable—BAECV [64]. If a disturbance did not intersect any of these datasets, then the causality was labeled as unknown [65]. We retrieved raster surfaces for the years between 1999 and 2016 (2015 and 2016 were retrieved from the Remap version), and extracted all pixels classified as unknown for each year. Once extracted, unknown disturbance areas were converted to polygons and cleaned (removed slivers and small polygons <1 ha). The complete 21-year fuel treatment polygon dataset (1999–2019) was used to estimate fuel treatment density per km^2^. First, we created a binary treatment raster (100-m cell size) from all fuel treatment polygons (1-a pixel received a treatment during any of the 21 years; 0-a pixel has not received any treatment), then we converted pixels with value 1 to points, and finally we applied the kernel density algorithm with a search radius of 1 km.

## 3. Results

### 3.1. Predictors and Random Forests Results

The most important predictors within the Random Forests classification method in terms of model error increase, after removing the variable, include the variables RBR, NDVI, bands 2 and 3 of the focal mean of PCA_sub_, and bands 1 and 2 of the focal STD of PCA_sub_. We found that most of the bands from PCA_div_ were correlated with the PCA_sub_ and as a result, they were not used in the analysis. NDVI and RBR were non-correlated with other bands and they were both used in the classification process. 

Validation of the Random Forests outputs for each of the five Sentinel-2 tiles for each year was performed with the OOB validation dataset of each tree (Table 2). The smallest average relative classification error was found for the period 2016–17 (2.6%, or a map accuracy of 97.4%), followed by 2018–19 (3.6%) and 2017–18 (4%). The root mean square error (RMSE) is again smaller for 2016–17 and highest for 2017–18 at 0.148 and 0.180, accordingly.

### 3.2. Temporal and Spatial Distrubution of Fuel Treatments

Fuel treatments of the 21-year studied period (1999–2019) covered approximately 153,000 ha in the four-county study area. The annual average sum of treated areas was 7565 ha (6820 ha when overlapping treatments of consecutive years were removed for the same treated polygon) for the period 1999–2016. Table 3 shows the distribution of treatment types per year. For 2017, we mapped 6238 ha of treatments; in 2018, we mapped approximately 5747 ha and in 2019, approximately 5186 ha. On average, 1850 ha were burned annually with prescribed fire for the period 1999–2016. The derived burned area surfaces (Section 2.9) captured a considerable number of prescribed fire units for 2017–2019, ranging from area estimates as low as 795 ha in 2017 to as high as 2342 ha in 2018. In 2019 approximately 1492 ha were attributed to prescribed fires. Most fuel treatments occurred during 2008 (9.3% of the total), followed by the year 2013 (7.6%), 2007 (7.2%), 2009 (6.7%) and 2001 (6.1%), while three consecutive years (2010–2012) received less than the 2.5% of the total treated area for the 21-year period (Table 3).

**Figure 5:**
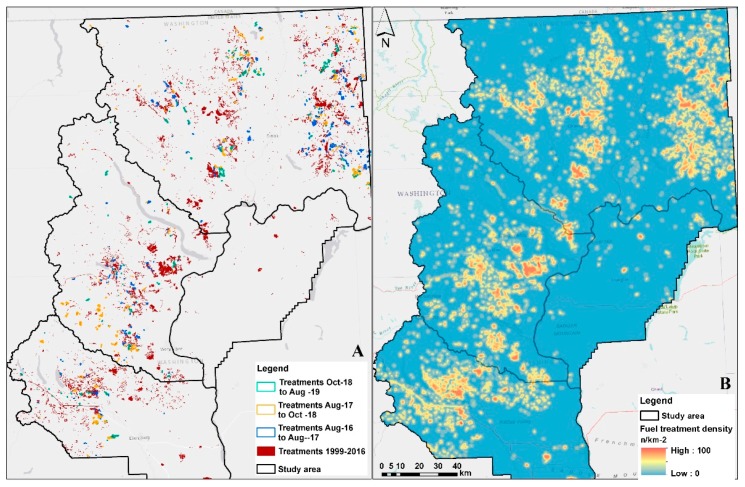
(**A**) Forest treatments for the three study periods (2016–17; 2017–18; 2018–19) with all other treatment locations for the years 1999–2016 (abrv: Oct = October, Aug = August); (**B**) density of all treatments for the period 1999–August 2019.

Of the four study area counties, half of the treated area occurred in Okanogan County, followed by Chelan County (28%), Kittitas County (20%) and Douglas County (1.3%) (Figure 5A). In Figure 5B, a kernel density map shows areas with a higher density of treatments over the 21-year study period, not accounting for overlapping treatments across the study period (i.e., areas where each pixel was treated at least once). The eastern and central parts of Okanogan County have large clusters of treatments, similar to the central parts of Chelan County and the north-central parts of Kittitas County where treatment density is high (Figure 5B).

**Figure 6:**
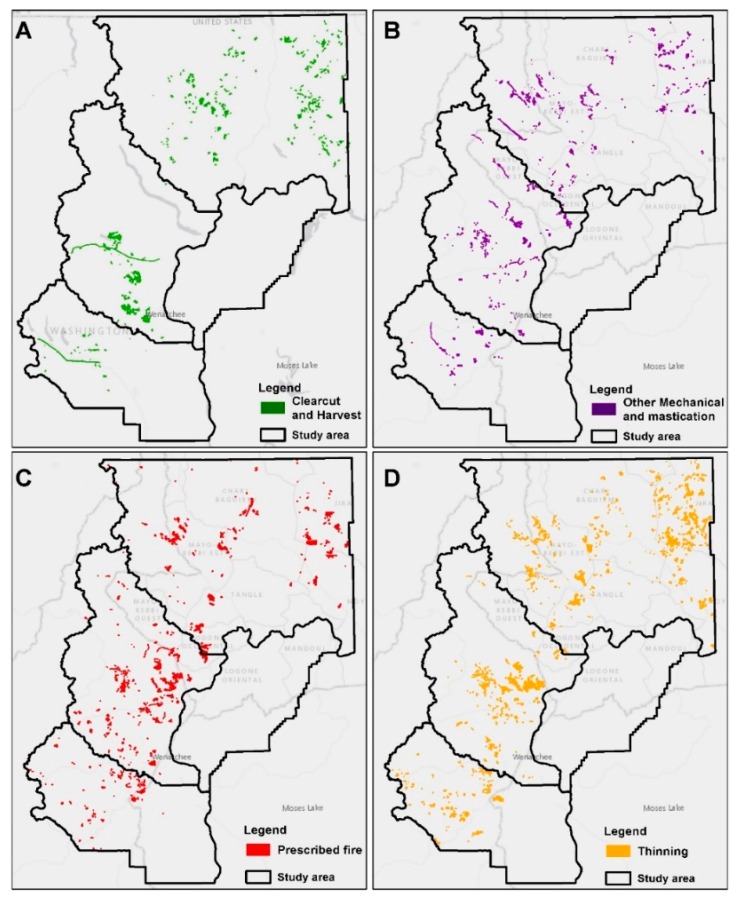
Spatial distribution of fuel treatment types across the study area for the period 1999–2019. (**A**) Clearcut and harvest; (**B**) Other mechanical and mastication; (**C**) Prescribed fire; (**D**) Thinning.

The southern parts of Chelan County and western Kittitas County receive the bulk of clearcut and harvest treatments (465 units of 4835 ha and 27 units of 500 ha, respectively), while the central and eastern parts of Okanogan County have a dense network of 470 treatments (18 ha average treatment size) covering 8560 ha (Figure 6A). Okanogan County received 9430 ha of mastication or other mechanical treatments on 725 units (average treatment size of 13 ha), followed by Chelan County with 5600 ha of treatments on a scattered pattern across the county (average treatment size of 13 ha on 425 units), and Kittitas County with 1790 ha of treatments on 98 units (average treatment size of 18 ha) (Figure 6B). Prescribed fire is an important fuel treatment practice in both Okanogan and Chelan Counties, which received 19,475 ha and 15,240 ha, respectively (Figure 6C). Chelan County has a smaller average size of treatment units (21 ha with 718 units) compared to Okanogan County (31 ha with 613 units). Kittitas County received 3160 ha of prescribed fire treatments on 204 units with 15 ha average size. The most important forest treatment activity in Okanogan County is thinning, with 18,515 ha of treatments on 1225 units with an average size of 15 ha (Figure 6D). Chelan County follows with 661 units of thinning, mostly allocated on the southeastern part of the county. In total, Chelan County received 11,500 ha of treatments (average treatment size of 17.5 ha). Finally, Kittitas County received 3980 ha of thinning treatments on 297 units with an average treatment size of 13.5 ha.

### 3.3. Accuracy Assessment

The “Accuracy Assessment” report depicts the number of correctly and incorrectly classified sampled observations (error matrix) and multiple statistics (Table 4, Table 5 and Table 6). Results revealed the highest overall accuracy (Overall) and the Chi-Square values for the 0.25 threshold. The standard error was also smaller for the first threshold. The same pattern was repeated for the other two pre- and post-treatment periods. This indicates that splitting the probabilistic output of Random Forests using a 0.25 threshold produces increased classification accuracy. Overall, classification accuracy was higher for 2016–17 (Table 4) and same for 2017–18 (Table 5) and 2018–19 (Table 6) for the 0.25 threshold (92.6%, 87.4% and 87.5% respectively). The average producers accuracy across the three periods for the positive value (1- location inside an actual fuel treatment) was 85.7%, 73.7% and 59.5% for the 0.25, 0.5 and 0.75 thresholds respectively, but at the same time for the negative value (0 - location outside an actual fuel treatment), the average producers accuracy was higher for all three thresholds, i.e., 92.6%, 97.5% and 99.2% respectively. An increasing threshold was better to locate true negative values, but the ability to locate true positive values was substantially decreasing. As expected, this independent accuracy assessment shows more classification error than the OOB relative classification error, given that treated areas were relatively scarce across the landscape (Table 2). 

**Figure 7:**
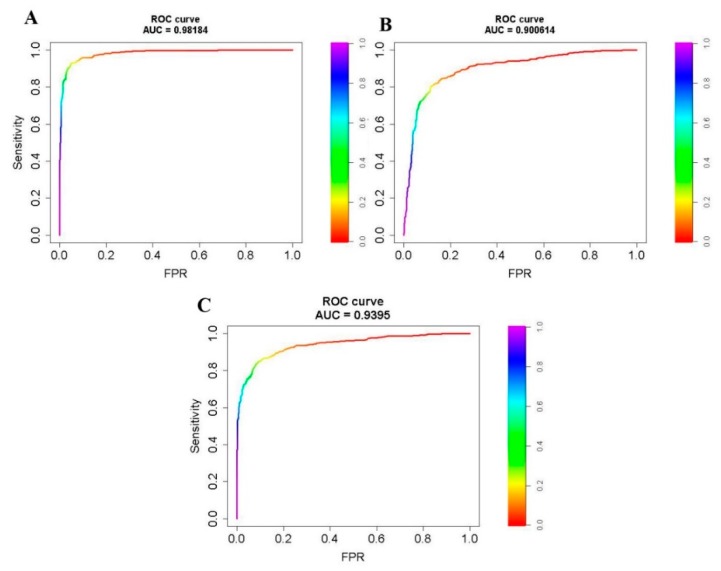
Receiver Operating Characteristics (ROC) curves derived from 1000-observation, independent validation dataset for the periods of: (**A**) 2016–2017; (**B**) 2017–2018; and (**C**) 2018–2019. Area Under Curve (AUC) denotes the overall model accuracy (values closer to 1—higher model accuracy). FPR on x-axis is the False Positive Rate (i.e., 1-specificity).

While our original sample observations did not have equal inclusion probabilities, the 30% treatment vs. 70% non-treatment threshold was closer to the prior distribution of treated and non-treated areas within our landscape as compared to our independent assessment. Using our independent sample, we assessed the more general condition when prior probabilities were equal across a hypothetical landscape. For each pre- and post-treatment time period, we produced ROC curves, where an AUC value near to 1 correlates with a good measure of separability (Figure 7). For all three periods, the AUC values were higher than 0.9, with years 2016–2017 having values close to 1 (i.e., 0.98).

## 4. Discussion

Our study describes a methodological approach to locate and map forest treatments (i.e., clearcut, thinning and prescribed fire) using free-of-charge data (Sentinel-2 satellite images, Landsat 8 Burned Area products, LANDFIRE data), with high classification accuracy and low implementation times. The 10-m resolution Sentinel-2 images were a key component of our methodology, since they provided a set of predictor variables able to capture the spectral changes between pre- and post-treatment images for clearcuts and thinnings, and allowed the creation of training datasets and the verification of outputs through visual interpretation. For example, these processes could not be accomplished using 30-m resolution Landsat images, especially for the scale of treatments we wanted to capture where each treated unit covers an area of a few hectares. Most similar previous research efforts were applied to tropical regions where disturbances and deforestation are extended and could be easily captured with coarser-scale remote sensing data.

Since our approach detected whether a pixel is treated or not, the post-processing classification between thinning and clearcut treatments (or other fuel treatment types) was a significant step to understand what happened within treated polygons. Sentinel-2 images were marginally adequate to detect between clearcuts and thinnings, especially for the larger treatments where we could clearly notice the reduction in forest cover and the post-treatment presence of trees after thinnings (Figure 4E,F). Furthermore, although the visual identification process of thinning treatments is prone to misclassification of natural disturbances such as tree mortality, insect attacks and weather damages that look similar to thinning, proximity to previous known treated sites increased the confidence of a polygon being a thinning. However, this was done without on-ground validation; thus, increasing the odds of misclassification. Defining the proper treatment type for the smaller sized polygons require on-ground knowledge of the study area and connections with locals that work in the area or know its management history. The Global Forest Change data [54] was a valuable dataset for both result validation and training datasets creation.

The efficiencies and flexibility of the Raster Utility, which enabled various analyses within this study, cannot be understated. Not only did it facilitate combining multiple spatial operations required to complete this study in one functional dataset, but also it greatly decreased the time and storage space traditionally needed to perform similar analyses. More specially, this utility allowed us to perform the required series of routine raster processes and more advanced functions for sampling, raster analysis, statistical modeling and machine learning (e.g., PCA and Random Forests) with its built-in functionality, avoiding the intermediate raster dataset creation. Consequently, complex and time-consuming analyses that would otherwise be computationally prohibitive to perform were easily and quickly incorporated into our described methodology. We expect that a user with moderate experience will need two to three weeks to complete a similar study with a 4-core desktop computer (i7-4770@3.2 GHz, 8 GB RAM) for an area similar to our study area extent (3.2 million ha).

Furthermore, the use of Random Forests to characterize and exploit structural characteristics in high dimensional data for the purposes of classification [66] directly within the ArcGIS environment through the Raster Utility toolbar proved to be a very robust and easy to use. These tools allowed us to calculate the statistical relationships between the predictor variables and sampled locations, evaluate and assess various relationships among predictor variables, and assess the accuracy of mapped forest treatments all within one environment, with an intuitive and easy-to-use graphical interface. Furthermore, model diagnostics helped us to determine the importance of certain variables through a series of operations that estimated the loss in prediction performance when particular variables were omitted from the training set. More importantly, though, resulting outputs produced from our study are very accurate and help to identify various types of treatments at fine spatial and temporal resolutions.

Potential users of this approach should carefully consider some important issues before replicating this study. First, the initial selection of satellite images that will be used to create the pre- and post-treatment image pairs. For example, we advise selecting images from the same satellite pass and images from similar time frames to minimize inconsistencies that may arise due to temporal phenological changes. Second, the most time-consuming process was the creation of samples to train the Random Forests models. Our described methodology required a visual inspection of the study area to identify potential sampling areas of actual forest treatments. Third, images can be challenging to retrieve at the desired temporal extent and with minimal cloud cover. North-Central Washington is an area that receives frequent precipitation, and even during the summer a large part of the study area is snow covered. This makes it difficult to locate cloud free images.

To answer our hypothesis, our approach can detect and map the spectral differences between mechanically treated and untreated lands with high accuracy (see Figure 4). The biggest challenge was to distinguish whether a resulting pixel from a Random Forests model with high probability of being treated was due to an actual treatment, agricultural lands changes, seasonal differences, burned area changes (natural- or human-caused), or satellite image features, such as shadows and clouds. All these reasons resulted in a high number of false positive polygons that we needed to manually remove from the dataset by visually inspecting each of them. This is common limitation for mapping efforts with remote sensing data. For example, the Multi-Index Integrated Change Analysis algorithm used in LANDFIRE [61] has omission and commission errors associated with the process, requiring a LANDFIRE mapping analyst to remove the incorrectly identified change using pre- and post-disturbance Landsat imagery. In addition, if treatment units occurred at times incongruent with image acquisition, our described methodology will not be able to detect and map those treatments. For example, if a treatment occurs after the initial acquisition of a pre-treatment image and the subsequent post-treatment image identifies a wildfire that overlapped that treatment, the treatment that occurred on the landscape will be spectrally covered by the wildfire event.

Most of the snow- or shadow-covered lands were non-forested, rugged or high-elevation landscapes, which helped us to remove falsely flagged polygons. The use of November Sentinel-2 images for the year 2018 caused many deciduous forests (hardwoods—Figure 1A) to be flagged as treated due to leaf drop and chlorophyll break down. We identified hardwood covered areas (mostly *Populus trichocarpa*) and removed all polygons whose tree cover density was unaltered over the 2019 satellite image. Despite using the LANDFIRE EVT mask to remove non-forested areas, parts of the landscape that were clearly agricultural lands were not captured by the mask. In such cases, we noticed that pixels were flagged since on the first year they were green and on the subsequent year they were plowed. Polygons with such characteristics were removed. The cases that were most difficult to discriminate were polygons located on burned lands. Despite removing all polygons within burned perimeters of the same period, we noted that many polygons appeared on lands that were burned one or two years prior to the sensing dates. This was evidence of a) delayed mortality, causing formerly green areas to lose their leaves, or b) salvage logging. We kept all polygons with clear evidence of salvage logging, i.e., proximity to new roads, straight polygon edges indicating human interference and neighboring past fuel treatments. 

Understanding previous forest activities may clarify how current forest conditions developed. For instance, if current conditions of a specific spatial extent meet a desired outcome related to forest or riparian health or habitat requirements for a given protected species, managers may be able to reconstruct the history of forest activities of that extent, such as frequency and type of forest treatment. Lessons learned from tracing these activities over time may inform management actions on similar forest extents to reach similar desired outcomes. 

A second way pinpointing details of previous treatments may inform future fuel treatments by elucidating jurisdictional patterns among past treatments. For example, if there are forest patches within a forest extent that have not yet reached a desired condition, understanding patterns among decision makers associated with those patches may point to issues or mechanisms that prevented them to facilitate the needed treatments, such as policy barriers or a lack of staffing capacity to complete the project. This may then allow decision makers to address these issues and subsequently execute future treatments more effectively. In sum, identifying details of previous treatments aids in linking outcomes (i.e., current forest conditions) to specific actions taken over time.

Finally, using these techniques and resulting datasets, forest managers have at their disposal consistent, spatially explicit information of treatment history that can be used to identify, justify, and optimize cross boundary prescriptions and treatments that can inform future land management decisions on landscape restoration to meet desired objectives. For example, spatial depictions of where, when, and the types of mechanical treatments that have been implemented across the landscape coupled with topography, the location and extent of previous fires, and existing vegetation condition, can be used together in a spatial overlay context to target opportunities to build fire suppression boundaries (e.g., [67]). Those boundaries could then be used to group and prioritize management prescriptions that reduce fuel loads, in turn reducing the impact of wildfire while meeting potential restoration objectives. Similarly, these same types of data, coupled with spatially explicit estimates of the forested condition (e.g., [68]), could be further used to monitor the effects of previous treatments and determine if those treatments were successful at meeting management objectives.

## 5. Conclusions

We presented a methodological procedure to locate and map forest treatments using data provided free-of-charge with high accuracy and low implementation times. Identifying the locations of clearcuts and thinnings was very straightforward and time efficient with the approach described. This approach worked well for the spatial extent of our study site, but may become challenging to manage at extremely large spatial extents. This makes it necessary to utilize tools such as the Raster Utility, that decreases the storage and processing power needed for data processing. We anticipate that the application of the methodology will enable researchers and other related land management endeavors to map past treatment units in a timely manner, both in the USA and throughout the world. The outputs of such replications could be used to inform natural resource research about the results of past management decisions, the associated processes, and the individuals or institutions involved or excluded from such decisions.

## Figures and Tables

**Table 1:** Sentinel image acquisition dates. S2A: Sentinel-2A; S2B: Sentinel-2B.

UFV	UFU	TFT	UGV	UGU
S2A-16/08/2016	S2A-16/08/2016	S2A-16/08/2016	S2A-16/08/2016	S2A-16/08/2016
S2B-26/08/2017	S2B-26/08/2017	S2A-02/07/2017	S2B-26/08/2017	S2B-26/08/2017
S2B-20/10/2018	S2B-20/10/2018	S2B-20/10/2018	S2B-20/10/2018	S2B-20/10/2018
S2B-06/08/2019	S2B-06/08/2019	S2A-06/08/2019	S2B-06/08/2019	S2B-06/08/2019

**Table 2:** Evaluation of Random Forests models predictive capabilities as estimated with the “out-of-bag” (i.e., validation) dataset. RMSE: Root Mean Square Error.

	2016–2017 Validation Errors					
Image id:	**TFT**	**UFU**	**UFV**	**UGU**	**UGV**	**Mean**
RMSE	0.127	0.141	0.146	0.185	0.142	0.148
Average Relative Error	0.043	0.052	0.046	0.078	0.048	0.053
Relative Classification Error	0.015	0.022	0.025	0.048	0.021	0.026
	**2017–2018 Validation Errors**					
RMSE	0.200	0.176	0.137	0.234	0.157	0.180
Average Relative Error	0.089	0.067	0.043	0.123	0.055	0.075
Relative Classification Error	0.045	0.034	0.019	0.068	0.035	0.040
	**2018–2019 Validation Errors**					
RMSE	0.181	0.133	0.115	0.215	0.211	0.171
Average Relative Error	0.082	0.045	0.036	0.113	0.100	0.075
Relative Classification Error	0.035	0.018	0.017	0.058	0.055	0.036

**Table 3:** Area of forest treatment types for each year of the 21-year study period, estimated in hectares. The column “Sum” summarizes the total treated area per year. It includes overlapping polygons for the period 1999-2014 (LANDFIRE Model Ready Events) of the same treated polygon. The column “Sum (no overlap)” removed overlapping treatments for the same treated polygon. CCut: Clearcut; Hvst: Harvesting; Mast: Mastication; Other Mech: Other Mechanical; PrFi: Prescribed Fire; Thin: Thinning; Unkn: Unknown.

Year	CCut	Hvst	Mast	Other Mech	PrFi	Thin	Unkn	Sum	Sum (No Overlap)
1999	31	1005	0	324	248	1492	4890	7990	7931
2000	0	183	0	528	235	1387	3449	5783	5319
2001	21	854	230	595	487	4760	2377	9325	8610
2002	58	662	229	294	407	966	3108	5724	5199
2003	99	490	38	588	700	702	3095	5712	5027
2004	116	988	30	401	417	2208	2976	7137	6705
2005	0	907	14	369	387	564	3424	5665	5177
2006	30	370	23	686	1670	1361	3799	7938	7396
2007	20	158	46	1052	5882	1876	2061	11,095	8477
2008	58	2015	192	1884	3588	4118	2474	14,330	13,088
2009	0	83	101	2742	4452	1063	1784	10,225	6744
2010	0	68	157	1415	441	825	725	3631	3163
2011	0	0	139	211	1063	917	1034	3363	3078
2012	6	247	100	100	1020	821	906	3200	2964
2013	0	0	325	1238	5426	1248	3401	11,639	10,553
2014	0	0	0	1259	2058	846	4371	8534	8431
2015	0	79	0	938	2005	931	3344	7297	7297
2016	0	29	0	567	2757	677	3554	7585	7585
2017	2381	n/a	n/a	n/a	795	3062	n/a	6238	6238
2018	1292	n/a	n/a	n/a	2342	2113	n/a	5747	5747
2019	1647	n/a	n/a	n/a	1492	2047	n/a	5186	5186
Sum	5758	8137	1626	15,191	37,871	33,985	50,774	153,342	139,915

**Table 4:** Accuracy Assessment report for the period 2016–17, as estimated with the Raster Utility tools based on a validation dataset of 1000 random samples (50% in each class). Random Forests results were split into treatment and no treatment pixels based on three thresholds (0.25, 0.5 and 0.75).

**Period: 2016–17**	**True Values**			**Threshold: 0.25**	Chi-square = 727.87; DF = 1; *p*-value = 2.59 × 10^−160^ Overall = 0.926 STE = 0.0165334462
Modeled Values	1	0	Total	User Accuracy	
1	450	24	474	94.9	
0	50	476	526	90.5	
Total	500	500			
Producer Accuracy	90.0	95.2			
**Period: 2016–17**	**True Values**			**Threshold: 0.5**	Chi-square = 623.52; DF = 1; *p*-value = 1.28 × 10^−137^ Overall = 0.887 STE = 0.0196266700
Modeled Values	1	0	Total	User Accuracy	
1	394	7	401	98.3	
0	106	493	599	82.3	
Total	500	500			
Producer Accuracy	78.8	98.6			
**Period: 2016–17**	**True Values**			**Threshold: 0.75**	Chi-square = 485.87; DF= 1; *p*-value = 1.12 × 10^−107^ Overall = 0.829 STE = 0.0224785626
Modeled Values	1	0	Total	User Accuracy	
1	332	3	335	99.1	
0	168	497	665	74.7	
Total	500	500			
Producer Accuracy	66.4	99.4			

**Table 5:** Accuracy Assessment report for the period 2017–18, as estimated with the Raster Utility tools based on a validation dataset of 1000 random samples (50% in each class). Random Forests results were split into treatment and no treatment pixels based on three thresholds (0.25, 0.5 and 0.75).

**Period: 2017–18**	**True Values**			**Threshold: 0.25**	Chi-square = 566.10; DF =1; *p*-value = 3.94 × 10^−125^ Overall = 0.874 STE = 0.0208652355
Modeled Values	1	0	Total	User Accuracy	
1	410	36	446	91.9	
0	90	464	554	83.8	
Total	500	500			
Producer Accuracy	82.0	92.8			
**Period: 2017–18**	**True Values**			**Threshold: 0.5**	Chi-square = 487.66; DF = 1; *p*-value = 4.59 × 10^−81^ Overall = 0.836 STE = 0.022535349
Modeled Values	1	0	Total	User Accuracy	
1	350	14	364	96.2	
0	150	486	636	76.4	
Total	500	500			
Producer Accuracy	70.0	97.2			
**Period: 2017–18**	**True Values**			**Threshold: 0.75**	Chi-square = 339.10; DF = 1; *p*-value = 9.99 × 10^−76^ Overall = 0.757 STE = 0.0239429508
Modeled Values	1	0	Total	User Accuracy	
1	261	4	265	98.5	
0	239	496	735	67.5	
Total	500	500			
Producer Accuracy	52.2	99.2			

**Table 6:** Accuracy Assessment report for the period 2018–19, as estimated with the Raster Utility tools based on a validation dataset of 1000 random samples (50% in each class). Random Forests results were split into treatment and no treatment pixels based on three thresholds (0.25, 0.5 and 0.75).

**Period: 2018–19**	**True Values**			**Threshold: 0.25**	Chi-square = 563.69; DF = 1; *p*-value = 1.32 × 10^−124^ Overall = 0.875 STE = 0.0208943592
Modeled Values	1	0	Total	User Accuracy	
1	426	51	477	89.3	
0	74	449	523	85.9	
Total	500	500			
Producer Accuracy	85.2	89.8			
**Period: 2018–19**	**True Values**			**Threshold: 0.5**	Chi-square = 509.17; DF = 1; *p*-value = 9.57 × 10^−113^ Overall = 0.846 STE = 0.0221384201
Modeled Values	1	0	Total	User Accuracy	
1	362	16	378	95.8	
0	138	484	622	77.8	
Total	500	500			
Producer Accuracy	72.4	96.8			
**Period: 2018–19**	**True Values**			**Threshold: 0.75**	Chi-square = 410.54; DF= 1; *p*-value = 2.79 × 10^−91^ Overall = 0.795 STE = 0.0235105510
Modeled Values	1	0	Total	User Accuracy	
1	300	5	305	98.4	
0	200	495	695	71.2	
Total	500	500			
Producer Accuracy	60.0	99.0			
